# Expansion, Maintenance, and Memory in NK and T Cells during Viral Infections: Responding to Pressures for Defense and Regulation

**DOI:** 10.1371/journal.ppat.1000816

**Published:** 2010-03-26

**Authors:** Christine A. Biron

**Affiliations:** Department of Molecular Microbiology and Immunology, Division of Biology and Medicine, Brown University, Providence, Rhode Island, United States of America; University of California San Francisco, United States of America

Immune responses are critical for defense. During primary infections, the antigen-specific CD8 T cells of the adaptive immune system are expanded from extremely low frequencies of cells and develop “memory” for heightened secondary responses. Innate natural killer (NK) cells, present at much higher frequencies, can also be expanded and develop memory. Subset NK and T cell proliferative responses are induced as a result of engagement of activator receptors for ligands expressed during particular viral infections, and the conditions are accompanied by stimulation of their antimicrobial functions. Increases in CD8 T cell numbers help optimize defense, but because of their higher basal frequencies, the need for increases in NK cell numbers is difficult to understand. The systems must have evolved under concurrent pressures for balance to limit damage from the immune responses themselves as well as to mediate defense against infection, however, as expanded NK and T cells present a threat for collateral damage. A recently defined function for an activating receptor in sustaining NK cells to produce interleukin (IL)-10 and regulate CD8 T cells is reviewed here in the context of T cell responses and memory. A rationale emerges for the role of proliferation in maintenance and regulation. The observations suggest that the immune system uses cell expansion to effectively deliver self-control as well as defense.

## Understanding of Functions for Endogenous Immune Responses to Viral Infections

The early understanding of innate immunity as non-specific and fast, and of antigen-specific adaptive immunity as slow but responsible for heightened recall or memory responses, was conceptually helpful but superficial. With the identification of pattern recognition sensors, their linkage to induction of particular innate cytokine responses, and the discernible consequences for shaping downstream innate and adaptive immunity, a generally accepted picture developed of a linear cascade of events in place to mediate defense during first infections. Long-lived immunity delivered by memory T cells was seen as an important end product (additional references online). The differentiation of innate and adaptive immunity is now being blurred with evidence demonstrating proliferation of NK cells of the innate immune system [1]–[3], and the suggestion in mouse systems that these cells can also contribute to memory responses [4]–[6]. Nevertheless, the assumption prevails that subset immune responses evolved to work in a positive feed-forward mechanism for activation of optimal antimicrobial defense.

There have been few fundamental challenges to mainstream thinking about the evolutionary pressures on the immune system, with the exception of the argument that no pressure exists for a memory immunity to boost antimicrobial effects [7]. If the first infection is controlled, why the need for “extra” immunity to fight re-infection? The case made in this argument is that responses leading to memory T cells are in place to fight infections in tissues. These conditions may require sustaining antigen-specific T cells for extended periods because first infections can persist. The possibility, however, that the immune system constituents have been selected based on functions other than promoting antimicrobial defense has not been thoroughly evaluated. The accumulating information on the disease and sometimes life-threatening consequences of subset immune responses to infection indicates that the pressure to self-regulate must have influenced the process. Innate cytokine storms contribute to pathogenesis during infections with murine cytomegalovirus (MCMV), human cytomegalovirus (HCMV), and human immunodeficiency virus (HIV); some of these cytokines, particularly IFN-γ and tumor necrosis factor (TNF), can also be produced by activated T cells to promote disease during infections with dengue virus, HIV, influenza virus, and hantavirus; and CD8 T cell–mediated injury occurs during infections with lymphocytic choriomeningitis virus (LCMV) and with hepatitis B and C viruses (HBV and HCV) (additional references online). The possibility exists, therefore, that there is pressure for selection of subset immune responses based on their negative regulatory functions. Such functions would be expected to be very important in the context of elevated numbers of NK and T cells under conditions of sustained infection and/or when their subsets have memory during re-infections. Here, a contribution to controlling immune responses to limit collateral damage resulting from heightened immunity might be as or more important as a contribution to sustaining antimicrobial defense. Because a need for cross-regulation to balance the multiple NK and T cell subsets responding would be required, the mechanisms would have to be distinct from those mediated at the level of a single cell (additional references online). Thus, there must be two pressures in place, one to protect from infection and another to protect from the immune responses when infections persist.

## Role of Activating Receptors—Expansion and Maintenance

Stimulation through the T cell receptors for antigen (TCRs) induces intracellular signaling pathways leading to cell division and activation (additional references online), and there are many examples of these CD8 T cell responses mediating defense during viral infections. The molecular mechanism in place of somatic gene rearrangements to generate diversity in the TCRs provides a level of protection against selection of pathogens failing to be detected by T cells of the adaptive immune system. This approach to solving the problem, however, results in very low frequencies of antigen-specific T cells at the time of primary exposure. Although CD8 T cells constitute about 10% of the white blood cells in peripheral compartments, the frequencies of cells specific for particular antigens prior to infection is <1 in 10^4^. Thus, obtaining sufficient numbers of these cells for fighting off infectious agents requires their expansion. NK cells also have activating receptors, mainly as products of their NK gene complexes, with Ly49 molecules in the mouse, KIR in the human, and NKG2 in both [8],[9]. In this situation, however, the genetic information is in germ line sequences, and there is variability in the diversity and numbers of genes inherited. When particular genes are present, the basal frequencies of NK cells expressing the activating receptor products are much higher than those of antigen-specific T cells. NK cells also represent approximately 5%–10% of the white blood cells in the peripheral compartments where they are found, but 20%–100% of these basally express particular activating receptors. NK cells can also express receptors delivering inhibitory signals, but experimental systems indicate that even when the proportions of these cells with reactivity to viral determinants are considered, the basal numbers of NK cells with the potential to respond are significantly higher than those of antigen-specific CD8 T cells. Thus, the requirement for NK cell expansion in defense is not obvious.

Nevertheless, engagement of NK cell–activating receptors stimulates intracellular signaling pathways overlapping with those accessed by TCRs (additional references online), and NK cell subsets expressing particular activating receptors are increased following a number of viral infections. Examples include expansion of the Ly49H subsets recognizing the MCMV ligand m157 during infections of mice with this virus [2], NKG2C/CD94 positive subsets in HCMV seropositive individuals [10], NKp30 subsets during chronic HCV infections [11], and KIR3DS1 positive subsets at times of acute HIV infections [12]. In contrast, certain conditions of viral infection induce dramatic reductions in NK cell functions, frequencies, and yields, including infections of humans with HIV [13],[14], HCV [15], and varicella zoster virus [16]. Examination of NK cell responses during MCMV infections of mice without the *Ly49h* gene has demonstrated a critical role for the Ly49H receptor not only in proliferation of the NK cell subsets, but also for NK cell maintenance under conditions of sustained infection [17]. If NK cell populations lack subsets with activating receptors for the viral ligand, their overall numbers decline to under pre-infection levels. This function for activating receptors had not been anticipated but may also be important in the context of maintaining antigen-specific T cells during sustained infection. In parallel to the situation of T cell stimulation through the TCR, the Ly49H molecule is also associated with the ability to develop memory NK cells [5]. Thus, activating receptors on NK and T cells might provide not only the machinery to induce proliferation and fight off infection, but also to support maintenance of the cells critically needed under conditions of extended viral infections. In the case of NK cells basally present at high frequencies, the contribution to cell maintenance may be more important than the contribution to increasing numbers.

## Maintenance for Regulation—IL-10 Production

In addition to their direct anti-viral effects, NK cells appear to have several mechanisms in place for mediating positive feed-forward effects on T cell responses to infections. They can produce IFN-γ to promote CD4 T cell differentiation into Th1 type cells [18]; regulate virus replication to reduce levels of type 1 interferons (IFN-αβ) and consequential negative effects on the availability of dendritic cells to enhance T cell responses [19]; and mediate killing of infected cells to promote antigen presentation for stimulation of T cells [20].

There is new evidence, however, that NK cells also can be activated to negatively regulate T cell responses because they can produce IL-10 [21]–[24]. First identified as a T cell product, a wide range of cells can be sources of IL-10 (additional references online). The cytokine is important for the regulation of T cell responses, and for protection against immune-mediated disease during infection (additional references online). NK cells make IL-10 in the context of chronic HCV infection [11] and, when Ly49H supports their maintenance, during uncontrolled infections with MCMV [17]. The NK cell IL-10 response during MCMV infection acts to limit the magnitude of the CD8 T cell response and protects from T cell–mediated disease. If the NK cells are not maintained, the regulation is lost with detrimental consequences. Interestingly, IL-10 is reported to play a role in limiting T cell responses to interfere with viral clearance rather than immunopathology during chronic LCMV infections [25],[26], and CD8 T cells can also be stimulated to make IL-10 in the context of influenza virus infections to protect against inflammatory disease [27]. Although the role for T cell–produced IL-10 in the control of NK cell responses remains to be demonstrated, it is likely to have an influence because NK cells respond to IL-10 [28]. The ability of memory CD8 T cells to produce IL-10 for protection against respiratory syncytial virus–induced lung injury in a model of vaccine-enhanced disease [29] suggests that this regulatory response will also be accessed during memory responses. Taken together, these observations indicate that the pathways in place to stimulate expansion and/or maintenance of NK and T cell numbers are linked to an ability to produce IL-10, and that production of the cytokine by either subset may provide auto- or cross-regulation. The association between maintenance and regulation makes it possible to access antimicrobial defense functions while limiting the overall magnitude of combined responses to protect from immune-mediated disease.

## Rethinking Acute and Memory Responses in Defense and Regulation

**Figure 1:**
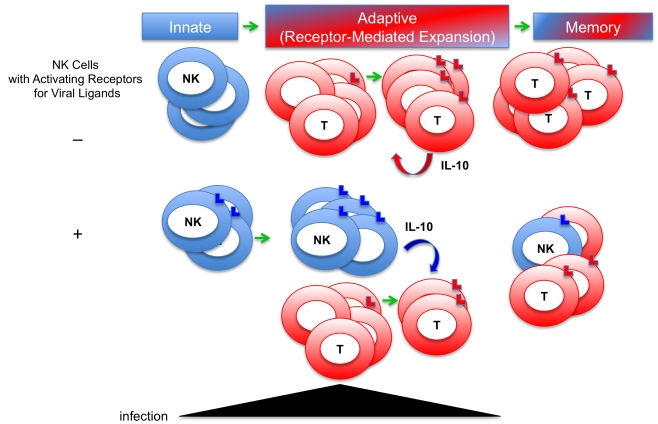
New model for defense and regulatory functions of immune responses to viral infections. Innate immune responses were first appreciated for their role in early antimicrobial defense. Adaptive responses, including T cell responses, were understood to take longer to develop during first infections because of the need to expand antigen-specific cell subsets from low frequency populations, and to be important for heightened defense, i.e., memory, during secondary infections. The evidence of innate NK cell proliferation and memory has blurred the distinction. Expansion of NK and T cells is a result of stimulation through activating receptors recognizing virus-induced ligands. New evidence indicates that a role for activating receptor-driven proliferation of NK cells is to promote their maintenance during viral infections, and that under conditions of extended infections, the cells produce IL-10 to regulate T cell responses. T cells can also be induced to produce IL-10 to control immune responses. Both NK and T cells can act to protect from immune-mediated disease. These observations underscore the need for evolutionary pressure to protect both from infection and from immune responses to infections.

The systems are complex and much remains to be learned, but the new picture emerging is that activating receptor-driven expansion of NK and T cells has important functions in addition to increasing the numbers of cell subsets equipped to mediate protection during acute infections (Figure 1). The presence of an activating receptor recognizing a viral ligand also confers the ability to maintain the population. Although not anticipated, such a function might have been predicted by the realization that in contrast to antigen-specific T cells, there should be less need to expand NK cell numbers for protection because the basal frequencies expressing particular activating receptors are high. The requirement for maintenance helps explain the conflicting reports of increases and decreases in NK cell proportions and numbers during infections by suggesting that in the absence of an activating receptor for a ligand induced by the virus, NK cells could be cleared. If they are gone, they can no longer contribute to defense or regulation. Sustaining or inducing memory NK cells through their activating receptors would make it possible to continue to access their antimicrobial or positive regulatory effects through other pathways, such as activation by cytokines [30]. However, under conditions of continued stimulation, these cells can also be accessed for their negative regulatory functions as demonstrated by their production of IL-10 to control adaptive T cell responses and protect from T cell–mediated disease. There are many other examples of IL-10 production regulating the magnitude of immune responses during infections, including production by CD8 T cells, and the function could be of value under conditions of secondary exposures because responding cells frequencies are increased. When taken together, the NK and T cell studies provide a strong rationale for rethinking the role of the relative pressures for defense and regulation in the evolution of the immune system. They indicate that in addition to being equipped to promote defense, the cells selected for expansion and maintenance have mechanisms to ensure cross-regulation under conditions of elevated numbers. The resulting new understanding underscores the importance, for survival, of protecting against both the infection and the immune response to infection.

## References

[1] Biron CA, Nguyen KB, Pien GC, Cousens LP, Salazar-Mather TP (1999). Natural killer cells in antiviral defense: function and regulation by innate cytokines.. Annu Rev Immunol.

[2] Dokun AO, Kim S, Smith HR, Kang HS, Chu DT (2001). Specific and nonspecific NK cell activation during virus infection.. Nat Immunol.

[3] Nguyen KB, Salazar-Mather TP, Dalod MY, Van Deusen JB, Wei XQ (2002). Coordinated and distinct roles for IFN-alpha beta, IL-12, and IL-15 regulation of NK cell responses to viral infection.. J Immunol.

[4] O'Leary JG, Goodarzi M, Drayton DL, von Andrian UH (2006). T cell- and B cell-independent adaptive immunity mediated by natural killer cells.. Nat Immunol.

[5] Sun JC, Beilke JN, Lanier LL (2009). Adaptive immune features of natural killer cells.. Nature.

[6] Cooper MA, Elliott JM, Keyel PA, Yang L, Carrero JA (2009). Cytokine-induced memory-like natural killer cells.. Proc Natl Acad Sci U S A.

[7] Zinkernagel RM (2002). On differences between immunity and immunological memory.. Curr Opin Immunol.

[8] Yokoyama WM, Seaman WE (1993). The Ly-49 and NKR-P1 gene families encoding lectin-like receptors on natural killer cells: the NK gene complex.. Annu Rev Immunol.

[9] Lanier LL (1998). NK cell receptors.. Annu Rev Immunol.

[10] Guma M, Angulo A, Lopez-Botet M (2006). NK cell receptors involved in the response to human cytomegalovirus infection.. Curr Top Microbiol Immunol.

[11] De Maria A, Fogli M, Mazza S, Basso M, Picciotto A (2007). Increased natural cytotoxicity receptor expression and relevant IL-10 production in NK cells from chronically infected viremic HCV patients.. Eur J Immunol.

[12] Alter G, Rihn S, Walter K, Nolting A, Martin M (2009). HLA class I subtype-dependent expansion of KIR3DS1+ and KIR3DL1+ NK cells during acute human immunodeficiency virus type 1 infection.. J Virol.

[13] Tarazona R, Casado JG, Delarosa O, Torre-Cisneros J, Villanueva JL (2002). Selective depletion of CD56(dim) NK cell subsets and maintenance of CD56(bright) NK cells in treatment-naive HIV-1-seropositive individuals.. J Clin Immunol.

[14] Azzoni L, Rutstein RM, Chehimi J, Farabaugh MA, Nowmos A (2005). Dendritic and natural killer cell subsets associated with stable or declining CD4+ cell counts in treated HIV-1-infected children.. J Infect Dis.

[15] Morishima C, Paschal DM, Wang CC, Yoshihara CS, Wood BL (2006). Decreased NK cell frequency in chronic hepatitis C does not affect ex vivo cytolytic killing.. Hepatology.

[16] Vossen MT, Biezeveld MH, de Jong MD, Gent MR, Baars PA (2005). Absence of circulating natural killer and primed CD8+ cells in life-threatening varicella.. J Infect Dis.

[17] Lee SH, Kim KS, Fodil-Cornu N, Vidal SM, Biron CA (2009). Activating receptors promote NK cell expansion for maintenance, IL-10 production, and CD8 T cell regulation during viral infection.. J Exp Med.

[18] Scharton TM, Scott P (1993). Natural killer cells are a source of interferon gamma that drives differentiation of CD4+ T cell subsets and induces early resistance to Leishmania major in mice.. J Exp Med.

[19] Robbins SH, Bessou G, Cornillon A, Zucchini N, Rupp B (2007). Natural killer cells promote early CD8 T cell responses against cytomegalovirus.. PLoS Pathog.

[20] Krebs P, Barnes MJ, Lampe K, Whitley K, Bahjat KS (2009). NK-cell-mediated killing of target cells triggers robust antigen-specific T-cell-mediated and humoral responses.. Blood.

[21] Brady J, Hayakawa Y, Smyth MJ, Nutt SL (2004). IL-21 induces the functional maturation of murine NK cells.. J Immunol.

[22] Maroof A, Beattie L, Zubairi S, Svensson M, Stager S (2008). Posttranscriptional regulation of II10 gene expression allows natural killer cells to express immunoregulatory function.. Immunity.

[23] Brockman MA, Kwon DS, Tighe DP, Pavlik DF, Rosato PC (2009). IL-10 is up-regulated in multiple cell types during viremic HIV infection and reversibly inhibits virus-specific T cells.. Blood.

[24] Deniz G, Erten G, Kucuksezer UC, Kocacik D, Karagiannidis C (2008). Regulatory NK cells suppress antigen-specific T cell responses.. J Immunol.

[25] Ejrnaes M, Filippi CM, Martinic MM, Ling EM, Togher LM (2006). Resolution of a chronic viral infection after interleukin-10 receptor blockade.. J Exp Med.

[26] Brooks DG, Trifilo MJ, Edelmann KH, Teyton L, McGavern DB (2006). Interleukin-10 determines viral clearance or persistence in vivo.. Nat Med.

[27] Sun J, Madan R, Karp CL, Braciale TJ (2009). Effector T cells control lung inflammation during acute influenza virus infection by producing IL-10.. Nat Med.

[28] Mocellin S, Panelli M, Wang E, Rossi CR, Pilati P (2004). IL-10 stimulatory effects on human NK cells explored by gene profile analysis.. Genes Immun.

[29] Stevens WW, Sun J, Castillo JP, Braciale TJ (2009). Pulmonary eosinophilia is attenuated by early responding CD8(+) memory T cells in a murine model of RSV vaccine-enhanced disease.. Viral Immunol.

[30] Lee SH, Miyagi T, Biron CA (2007). Keeping NK cells in highly regulated antiviral warfare.. Trends Immunol.

